# Survival prognostic nomogram for young metastatic non-small cell lung cancer: a study of the US SEER database and a Chinese cohort

**DOI:** 10.3389/fonc.2025.1502253

**Published:** 2025-02-14

**Authors:** Yu Li, Lei Cao, Yawen Ding, Lei Liu, Yonggang Zhu, Feng Cao

**Affiliations:** ^1^ Department of Radiation Oncology, The Fourth Hospital of Hebei Medical University, Shijiazhuang, China; ^2^ Department of Geriatric Respitatory, Hebei General Hospital, Shijiazhuang, China; ^3^ Clinical Laboratory, The Fourth Hospital of Hebei Medical University, Shijiazhuang, China; ^4^ Department of Thoracic Surgery, The Fourth Hospital of Hebei Medical University, Shijiazhuang, China

**Keywords:** young patients, metastatic NSCLC, nomogram, overall survival, prognosis

## Abstract

**Objective:**

Young patients diagnosed with non-small cell lung cancer (NSCLC) present unique clinical, pathological, and genetic features, resulting in a highly heterogeneous patient population. The current TNM staging system is insufficient for accurately predicting their prognosis. This study aims to develop a nomogram model for survival prediction in young patients with metastatic NSCLC at initial diagnosis and further verify the effectiveness of the model.

**Methods:**

This study enrolled 961 young patients diagnosed with metastatic NSCLC in the Surveillance, Epidemiology, and End Results (SEER) database between 2010 and 2017. The patients were allocated into a training cohort (*n* = 673) and an internal validation cohort (*n* = 288). An additional 215 patients from the Fourth Hospital of Hebei Medical University were included as a Chinese external validation cohort. Univariate and multivariate Cox regression analyses were conducted in the training cohort to identify independent risk factors influencing survival, which were used to develop a nomogram model. The model’s effectiveness was evaluated using C-index, calibration curve, receiver operating characteristic (ROC) curve, decision curve analysis (DCA) curve, and Kaplan–Meier survival curve.

**Results:**

The multifactorial Cox regression model identified eight independent risk factors influencing overall survival (OS): race, marital status, histological type, T stage, N stage, liver metastasis, chemotherapy, and radiotherapy (all *P* < 0.05). These factors were incorporated into the nomogram, which achieved a C-index of 0.673 [95% confidence interval (CI) = 0.661–0.685]. The nomogram exhibited excellent prognostic value in both internal (C-index = 0.662, 95% CI = 0.643–0.681) and external (C-index = 0.724, 95% CI = 0.702–0.746) validation cohorts. In addition, calibration curves for 0.5-,1-, 2-, 3-, and 5-year OS probabilities showed close agreement between predicted and observed survival outcomes across various time points. Additionally, ROC curve analysis and Kaplan–Meier curves highlighted the robust discriminatory power of the model based on survival outcomes. Moreover, the DCA analysis revealed that the incremental net benefit of this model was significantly superior to that of the TNM staging system alone.

**Conclusions:**

A nomogram model has been developed and validated to accurately predict the OS of young patients with metastatic NSCLC at initial diagnosis, demonstrating superior performance compared to the traditional TNM staging system. This model offers valuable guidance for precise predictions and making rational treatment decisions in clinical practice.

## 1 Introduction

Lung cancer remains the leading cause of cancer mortality worldwide (1, 2). Non-small cell lung cancer (NSCLC) accounts for approximately 85% of all lung cancer cases; incidence of NSCLC per 100,000 in the United States has shown a positive trend, dropping from 46.4 in 2010 to 40.9 in 2017 overall. Nevertheless, the incidence of metastatic NSCLC at diagnosis has decreased slightly from 21.7 to 19.6 during the same period (3–5). Metastatic NSCLC demonstrates considerable heterogeneity, even within the same TNM stage, leading to varying survival outcomes, which poses a challenging issue in clinical decision-making. Due to the low incidence of lung cancer in young people, screening and early detection strategies often focus more on older populations with higher risk. Most previous studies have been single-center, small-sample, and retrospective studies, leading to a lack of consensus on the prognosis of young lung cancer. Some studies suggest that young and elderly lung cancer patients have similar prognosis, while others propose that younger patients may have better prognosis despite advanced stage due to their superior physical condition and ability to tolerate more treatment options (6–9).

Nomograms are widely utilized tools for prognostic estimation in the fields of oncology and medicine. By integrating a variety of prognostic and determinant variables, nomograms can generate an individualized numerical probability of clinical events. This capability addresses the need for models that combine biological and clinical factors, aligning with the goals of personalized medicine and enhancing the precision of patient care (10, 11). Previous studies have indicated that nomograms provide more accurate survival predictions than traditional TNM staging in early stage NSCLC (12, 13). However, there has been no prognostic model specifically developed or validated for young patients with metastatic NSCLC at initial diagnosis. There is an urgent need for research on epidemiological characteristics, treatment options, and prognostic evaluation of young patients with metastatic NSCLC.

This study utilized the Surveillance, Epidemiology, and End Results (SEER) database of the National Cancer Institute of the United States (14) to investigate the clinical features and prognostic factors of young patients with metastatic NSCLC at initial diagnosis. It also introduces and evaluates the validity of a novel prognostic nomogram model, aiming to enhance understanding, clarify prognostic factors, and optimize treatment approaches for this demographic.

## 2 Methods

### 2.1 Patient selection

**Figure 1:**
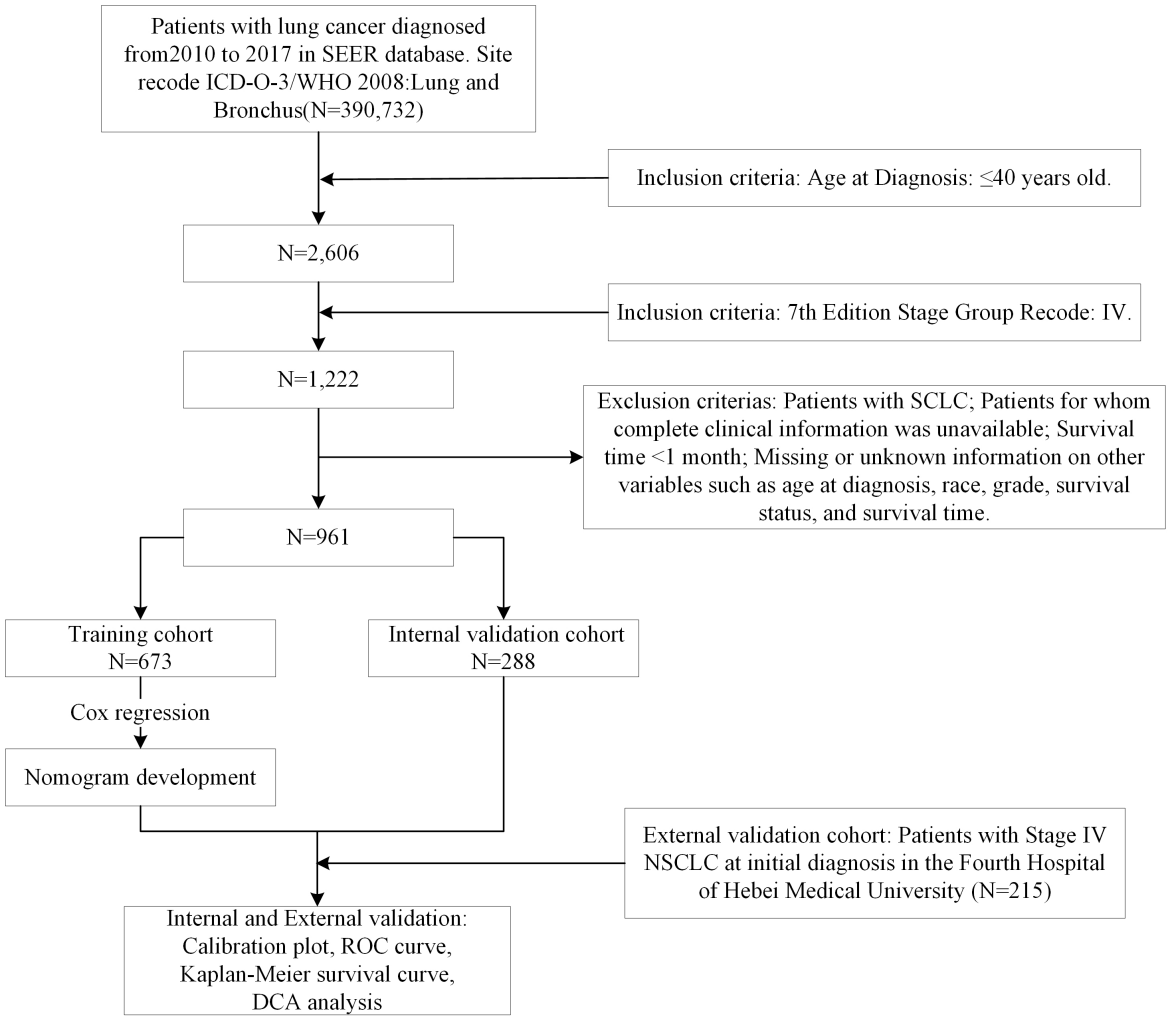
Design flowchart of this study.

Patients were selected from 17 population-based cancer registries within the SEER database (http://seer.cancer.gov/). The SEER*Stat program v8.4.3 (seer.cancer.gov/seerstat) facilitated the extraction of lung cancer patient data. Total of 961 patients diagnosed with metastatic NSCLC from 2010 to 2017, aged ≤40 years, were included based on specific criteria (1): diagnostic year between 2010 and 2017 (2), ICD-O-3/WHO 2008 site recode for lung and bronchus (3), 7th Edition Stage Group Recode: IV, and (4) age at diagnosis: ≤40 years. Exclusion criteria were (1) the pathological type was SCLC (2), incomplete clinical information (3), survival time of less than one month, and (4) missing or unknown data on variables such as age at diagnosis, race, histological type, survival status, and survival time (**Figure 1**).

These patients were randomly divided into training and internal validation cohorts with a bootstrapping technique in 7:3 ratio. Additionally, 215 patients with metastatic NSCLC initial diagnosed in youth from January 2013 to December 2023 at the Fourth Hospital of Hebei Medical University served as an external validation cohort. Inclusion criteria for this cohort were (1) NSCLC confirmed by histopathology (2), initial diagnosis of stage IV at the Fourth Hospital of Hebei Medical University (3), age ≤ 40 years, and (4) complete general examination and clinicopathological data. Exclusion criteria included (1) a history of other malignant tumors (2); hematological, immune system, or infectious diseases (3); prior anti-tumor treatments (4); discontinuation of treatment during hospitalization; or (5) survival time of less than 1 month.

### 2.2 Study variables

The following variables were extracted: “Age recode with <1 year olds,” “Sex,” “Race recode (White, Black, Other),” “Marital status at diagnosis,” “Laterality,” “Primary Site–labeled,” “ Histologic Type ICD-O-3,” “Derived AJCC T, 7th ed (2010–2015).,” “Derived AJCC N, 7th ed (2010–2015).,” “Derived AJCC M, 7th ed (2010–2015).,” “Derived AJCC T, 7th ed (2016–2017).,” “Derived AJCC N, 7th ed (2016–2017).,” “Derived AJCC M, 7th ed (2016–2017).,” “SEER Combined Mets at DX-bone (2010+),” “SEER Combined Mets at DX-brain (2010+),” “SEER Combined Mets at DX-liver (2010+),” “SEER Combined Mets at DX-lung (2010+),” “Chemotherapy recode,” “Radiation recode,” “Vital status recode,” “Survival months.” In the analysis, patients were classified by age using a recode for ages ≤ 40 years. Continuous variables like age at diagnosis were converted into categorical variables, grouping patients into ≤35 years old or >35 years old.

### 2.3 Nomogram development

Using univariate Cox proportional hazards regression model, potential risk factors in the training cohort were analyzed to determine the hazard ratio (HR) and 95% CI for each risk factor. Significant risk factors with *P* < 0.05 were subsequently included in the Cox multivariable regression analysis. Statistically significant factors (*P* < 0.05) in the multivariable analysis were utilized to construct a nomogram for survival probability of young metastatic NSCLC patients at 0.5-, 1-, 2-, 3-, and 5-year time points.

### 2.4 Nomogram validation

The discriminative ability and calibration of the chart were assessed using an internal training cohort and an external validation cohort. Model performance was evaluated based on the C-index (ranging from 0.5 for no predictive discriminative ability to 1.0 for perfect discriminative ability), with a value greater than 0.7 indicating reliable discriminative ability. The calibration curve compares predicted probability with observed results, ideally aligning along the 45° line to indicate perfect calibration. The receiver operating characteristic (ROC) curve and area under the curve (AUC) values at 0.5, 1, 2, 3, and 5 years quantify the model’s discriminative ability. The clinical utility of the chart was compared with the TNM staging system using decision curve analysis (DCA). Kaplan–Meier curves were used to analyze survival differences between high-risk and low-risk groups identified by the chart.

### 2.5 Statistical analysis

Statistical analyses were conducted with SPSS version 27.0 and R software version 4.3.2, with the determination of optimal thresholds for variables like body mass index (BMI), neutrophil to lymphocyte ratio (NLR), lymphocyte to monocyte ratio (LMR), and platelet to lymphocyte ratio (PLR) were conducted using X-Tile software. Categorical variables were assessed using chi-square test or Fisher’s exact test, while survival curves were compared using the log-rank test. Univariate and multivariate Cox regression analyses identified factors significantly associated with outcomes, with *P* values < 0.05 deemed statistically significant. Survival plots were generated using R software, and model performance was evaluated utilizing C index, calibration curves, ROC curves, DCA curves, and Kaplan–Meier curves.

## 3 Results

### 3.1 Demographic and clinicopathological characteristics

In the present study, a total of 390,732 cases of lung cancer were reported in the SEER database from 2010 to 2017. Among these cases, 2,606 patients were aged 40 years or younger, accounting for approximately 0.67% of all lung cancer patients. A total of 1,222 patients out of 2,606 cases were diagnosed with stage IV lung cancer, representing 46.9% of all lung cancers in young patients. Among these, 961 patients were diagnosed with NSCLC. All analytical variables included in the SEER database are listed in **Table 1**. A total of 961 patients were randomly divided into the training cohort (*n* = 673) and the internal validation cohort (*n* = 288). No statistically significant differences were found between the two cohorts in terms of age, gender, race, marital status, side, primary site, histological type, T stage, N stage, M stage, presence of bone, brain, liver, lung metastasis, chemotherapy, and radiotherapy.

**Table 1:** Demographics and clinicopathological characteristics of the training and internal validation cohort.

Variables	Training cohort (*N* = 673)	Internal validation cohort (*N* = 288)	*P*-value
Age			0.095
≤ 35	274 (40.7%)	134 (46.5%)	
> 35	399 (59.3%)	154 (53.5%)	
Gender			0.540
Male	329 (48.9%)	147 (51.0%)	
Female	344 (51.1%)	141 (49.0%)	
Race			0.163
White	451 (67.0%)	182 (63.2%)	
Black	101 (15.0%)	39 (13.5%)	
Other^1^	121 (18.0%)	67 (23.3%)	
Marital status			0.904
Married	350 (52.0%)	151 (52.4%)	
Others^2^	323 (48.0%)	137 (47.6%)	
Side			0.077
Left	261 (38.8%)	110 (38.2%)	
Right	355 (52.7%)	165 (57.3%)	
Bilateral	57 (8.5%)	13 (4.5%)	
Primary site			0.610
Main bronchus	29 (4.3%)	8 (2.8%)	
Upper lobe	262 (38.9%)	125 (43.4%)	
Middle lobe	36 (5.3%)	16 (5.6%)	
Lower lobe	190 (28.2%)	72 (25.0%)	
Overlapping lesion	14 (2.1%)	4 (1.4%)	
Lung NOS	142 (21.1%)	63 (21.9%)	
Histological type			0.587
Adenocarcinoma	494 (73.4%)	211 (73.3%)	
Squamous	130 (19.3%)	51 (17.7%)	
Others^3^	49 (7.3%)	26 (9.0%)	
T stage			0.094
T0	7 (1.0%)	2 (0.7%)	
T1	67 (10.0%)	41 (14.2%)	
T2	144 (21.4%)	60 (20.8%)	
T3	165 (24.5%)	83 (28.8%)	
T4	290 (43.1%)	102 (35.4%)	
N			0.097
N0	135 (20.1%)	43 (14.9%)	
N1	55 (8.2%)	35 (12.2%)	
N2	280 (41.6%)	121 (42.0%)	
N3	203 (30.2%)	89 (30.9%)	
M			0.778
M1a	143 (21.2%)	65 (22.6%)	
M1b	509 (75.6%)	216 (75.0%)	
M1NOS	21 (3.1%)	7 (2.4%)	
Bone metastasis			0.528
No	359 (53.3%)	160 (55.6%)	
Yes	314 (46.7%)	128 (44.4%)	
Brain metastasis			0.929
No	442 (65.7%)	190 (66.0%)	
Yes	231 (34.3%)	98 (34.0%)	
Liver metastasis			0.978
No	531 (78.9%)	227 (78.8%)	
Yes	142 (21.1%)	61 (21.2%)	
Lung metastasis			0.488
No	433 (64.3%)	192 (66.7%)	
Yes	240 (35.7%)	96 (33.3%)	
Chemotherapy			0.065
No	128 (19.0%)	41 (14.2%)	
Yes	545 (81.0%)	247 (85.8%)	
Radiotherapy			0.468
No	310 (46.1%)	140 (48.6%)	
Yes	363 (53.9%)	148 (51.4%)	

Other^1^: American Indian, AK Native, Asian or Pacific Islander.

Others^2^: Unmarried or Domestic Partner, Single (never married), Widowed, Divorced, Separated

Others^3^: Mucoepidermoid carcinoma, Mucinous adenocarcinoma, Synovial sarcoma.

Additionally, a cohort of 215 patients diagnosed with metastatic NSCLC from the Fourth Hospital of Hebei Medical University formed the external validation cohort. A comparison of the demographic and clinicopathological characteristics between the SEER cohort and the external validation cohort is presented in **Table 2**. Notably, all patients in the external validation cohort were Chinese, while they were categorized as “other” in the SEER database. The proportion of married patients was significantly higher in the external validation cohort than in the SEER cohort (*P* < 0.05), as were the proportions of bilateral lesions, adenocarcinoma, T1 stage, N3 stage, M1a stage, bone metastasis, and patients not receiving chemotherapy or radiotherapy (all *P* < 0.05). No significant differences were observed between the two cohorts in terms of age, gender, primary site, brain metastasis, liver metastasis, and lung metastasis (all *P* > 0.05). These discrepancies underscore the robustness of the external validation process.

**Table 2:** Demographics and clinicopathological characteristics of the external validation and SEER cohort.

Variables	External cohort (*N* = 215)	SEER cohort (*N* = 961)	*P*-value
Age			0.226
≤ 35	101 (47.0%)	408 (42.5%)	
> 35	114 (53.0%)	553 (57.5%)	
Gender			0.854
Male	105 (48.8%)	476 (49.5%)	
Female	110 (51.2%)	485 (50.5%)	
Race			< 0.001
White	0 (0%)	633 (65.9%)	
Black	0 (0%)	140 (14.6%)	
Other^1^	215 (100%)	188 (19.6%)	
Marital status			< 0.001
Married	188 (87.4%)	501 (52.1%)	
Others^2^	27 (12.6%)	460 (47.9%)	
Side			< 0.001
Left	73 (34.0%)	371 (38.6%)	
Right	84 (39.1%)	520 (54.1%)	
Bilateral	58 (27.0%)	70 (7.3%)	
Primary site			0.333
Main bronchus	12 (5.6%)	37 (3.9%)	
Upper lobe	93 (43.3%)	387 (40.3%	
Middle lobe	15 (7.0%)	52 (5.4%)	
Lower lobe	59 (27.4%)	262 (27.3%)	
Overlapping lesion	3 (1.4%)	18 (1.9%)	
Lung NOS	33 (15.3%)	205 (21.3%)	
Histological type			< 0.001
Adenocarcinoma	205 (95.3%)	705 (73.4%)	
Squamous	7 (3.3%)	181 (18.8%)	
Others^3^	3 (1.4%)	75 (7.8%)	
T stage			< 0.001
T0	0 (0%)	9 (0.9%)	
T1	46 (21.4%)	108 (11.2%)	
T2	50 (23.3%)	204 (21.2%)	
T3	44 (20.5%)	248 (25.8%)	
T4	75 (34.9%)	392 (40.8%)	
N stage			< 0.001
N0	13 (6.0%)	178 (18.5%)	
N1	47 (21.9%)	90 (9.4%)	
N2	34 (15.8%)	401 (41.7%)	
N3	121 (56.3%)	292 (30.4%)	
M stage			< 0.001
M1a	73 (34.0%)	208 (21.6%)	
M1b	142 (66.0%)	725 (75.4%)	
M1NOS	0 (0%)	28 (2.9%)	
Bone metastasis			0.013
No	96 (44.7%)	519 (54.0%)	
Yes	119 (55.3%)	442 (46.0%)	
Brain metastasis			0.465
No	147 (68.4%)	632 (65.8%)	
Yes	68 (31.6%)	329 (34.2%)	
Liver metastasis			0.410
No	175 (81.4%)	758 (78.9%)	
Yes	40 (18.6%)	203 (21.1%)	
Lung metastasis			0.915
No	139 (64.7%)	625 (65.0%)	
Yes	76 (35.3%)	336 (35.0%)	
Chemotherapy			0.007
No	55 (25.6%)	169 (17.6%)	
Yes	160 (74.4%)	792 (82.4%)	
Radiotherapy			< 0.001
No	156 (72.6%)	450 (46.8%)	
Yes	59 (27.4%)	511 (53.2%)	
Immunotherapy			NA
No	195 (90.7%)	NA	
Yes	20 (9.3%)	NA	
Targeted therapy			NA
No	78 (36.3%)	NA	
Yes	137 (63.7%)	NA	

Other^1^: American Indian, AK Native, Asian or Pacific Islander.

Others^2^: Unmarried or Domestic Partner, Single (never married), Widowed, Divorced, Separated

Others^3^: Mucoepidermoid carcinoma, Mucinous adenocarcinoma, Synovial sarcoma.

### 3.2 Univariate and multivariate analysis of the OS in the training cohort

The median survival time of these 961 patients was 14 months. 0.5-, 1-, 2-, 3-, and 5-year overall survival (OS) were 74.3%, 55.8%, 35.7%, 26.5%, and 14.7%, respectively. The 961 young patients with metastatic NSCLC from the SEER database in this study were randomly allocated into the training group and the internal validation group. We utilized the Cox proportional hazards regression model to conduct an analysis of the OS within the training cohort (*n* = 673). Univariate analysis revealed significant associations between gender, race, marital status, primary site, histological type, T stage, N stage, liver metastasis, chemotherapy, and radiotherapy with OS (*P* < 0.05). Conversely, age, side, M stage, bone metastasis, brain metastasis, and lung metastasis did not show significant associations with OS (*P* > 0.05) (**Table 3**). The multivariate model following stepwise regression demonstrated that race, marital status, histological type, T stage, N stage, liver metastasis, chemotherapy, and radiotherapy were significantly associated with survival (all *P* < 0.05).

**Table 3:** Univariate and multivariate Cox regression analysis of various factors for predicting OS in the training cohort.

Variables	Univariate analysis		Multivariate analysis	
	HR (95% CI)	*P*-value	HR (95% CI)	*P*-value
Age		0.076		
≤ 35	Reference		NA	
> 35	1.168 (0.984–1.388)		NA	
Gender		0.004		0.921
Male	Reference		Reference	
Female	0.783 (0.662–0.925)		0.921 (0.774–1.096)	
Race		0.008		0.014
White	Reference		Reference	
Black	1.005 (0.792–1.275)		0.894 (0.689–1.144)	
Other^1^	0.711 (0.567–0.893)		0.707 (0.560–0.893)	
Marital status		< 0.001		0.008
Married	Reference		Reference	
Others^2^	1.354 (1.145–1.601)		1.269 (1.064–1.514)	
Side		0.888		
Left	Reference		NA	
Right	0.981 (0.822–1.169)		NA	
Bilateral	0.923 (0.666–1.281)		NA	
Primary site		< 0.001		0.1
Main bronchus	1.175 (0.758–1.821)		1.383 (0.882–2.168)	
Upper lobe	0.478 (0.261–0.877)		0.630 (0.340–1.168)	
Middle lobe	0.897 (0.573–1.404)		1.181 (0.747–1.865)	
Lower lobe	2.078 (1.061–4.068)		2.517 (1.271–4.984)	
Overlapping lesion	1.216 (0.772–1.916)		1.572 (0.987–2.503)	
Lung NOS	Reference		Reference	
Histological type		< 0.001		0.036
Adenocarcinoma	Reference		Reference	
Squamous	2.052 (1.509–2.791)		1.502 (1.088–2.072)	
Others^3^	1.128 (0.985–1.508)		1.125 (0.906–1.396)	
T stage				
T0	Reference	< 0.001	Reference	< 0.001
T1	4.174 (1.013–17.176)		5.796 (1.395–24.087)	
T2	4.828 (1.192–19.554)		6.151 (1.503–25.174)	
T3	5.583 (1.381–22.571)		6.339 (1.550–25.920)	
T4	6.913 (1.717–27.834)		9.158 (2.225–37.282)	
N stage		< 0.001		0.001
N0	Reference		Reference	
N1	1.139 (0.929–1.837)		1.405 (0.978–2.017)	
N2	1.557 (1.229–1.971)		1.540 (1.198–1.979)	
N3	(1.171–1.935)		1.677 (1.287–2.186)	
M stage		0.157		
M1a	Reference		NA	
M1b	1.223 (0.992–1.506)		NA	
M1NOS	1.170 (0.702–1.949)			
Bone metastasis		0.067	NA	
No	Reference			
Yes	1.170 (0.989–1.383)		NA	
Brain metastasis		0.133	NA	
No	Reference			
Yes	0.873 (0.733–1.041)		NA	
Liver metastasis		< 0.001		< 0.001
No	Reference		Reference	
Yes	1.519 (1.244–1.854)		1.474 (1.202–1.806)	
Lung metastasis		0.405	NA	
No	Reference			
Yes	1.077 (0.905–1.281)		NA	
Chemotherapy		<0.001		< 0.001
No	Reference		Reference	
Yes	0.617 (0.500–0.761)		0.531 (0.424–0.666)	
Radiotherapy		<0.001		< 0.001
No	Reference		Reference	
Yes	1.343 (1.134–1.591)		1.445 (1.212–1.724)	

Other^1^: American Indian, AK Native, Asian or Pacific Islander.

Others^2^: Unmarried or Domestic Partner, Single (never married), Widowed, Divorced, Separated

Others^3^: Mucoepidermoid carcinoma, Mucinous adenocarcinoma, Synovial sarcoma.

### 3.3 Training cohort survival

**Figure 2:**
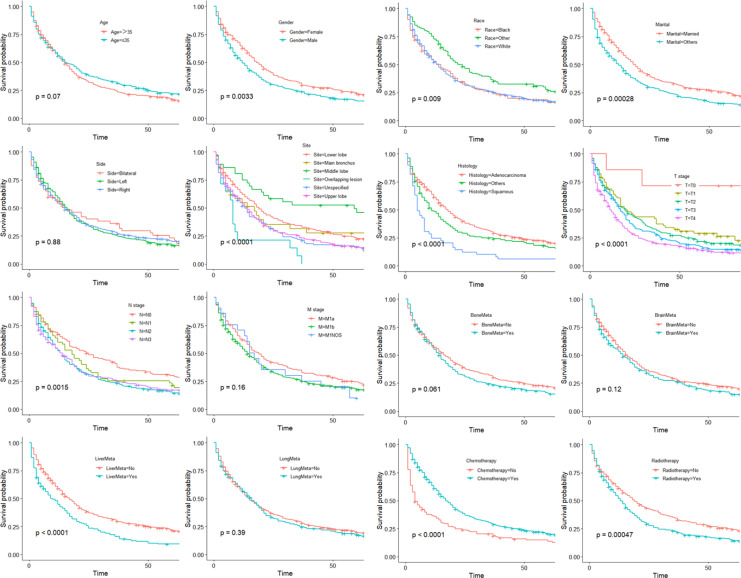
Kaplan–Meier curves of OS in the training cohort.

Survival curves were generated using the Kaplan–Meier method for the 16 variables (**Figure 2**).

### 3.4 Prognosis model of young patients with metastatic NSCLC

**Figure 3:**
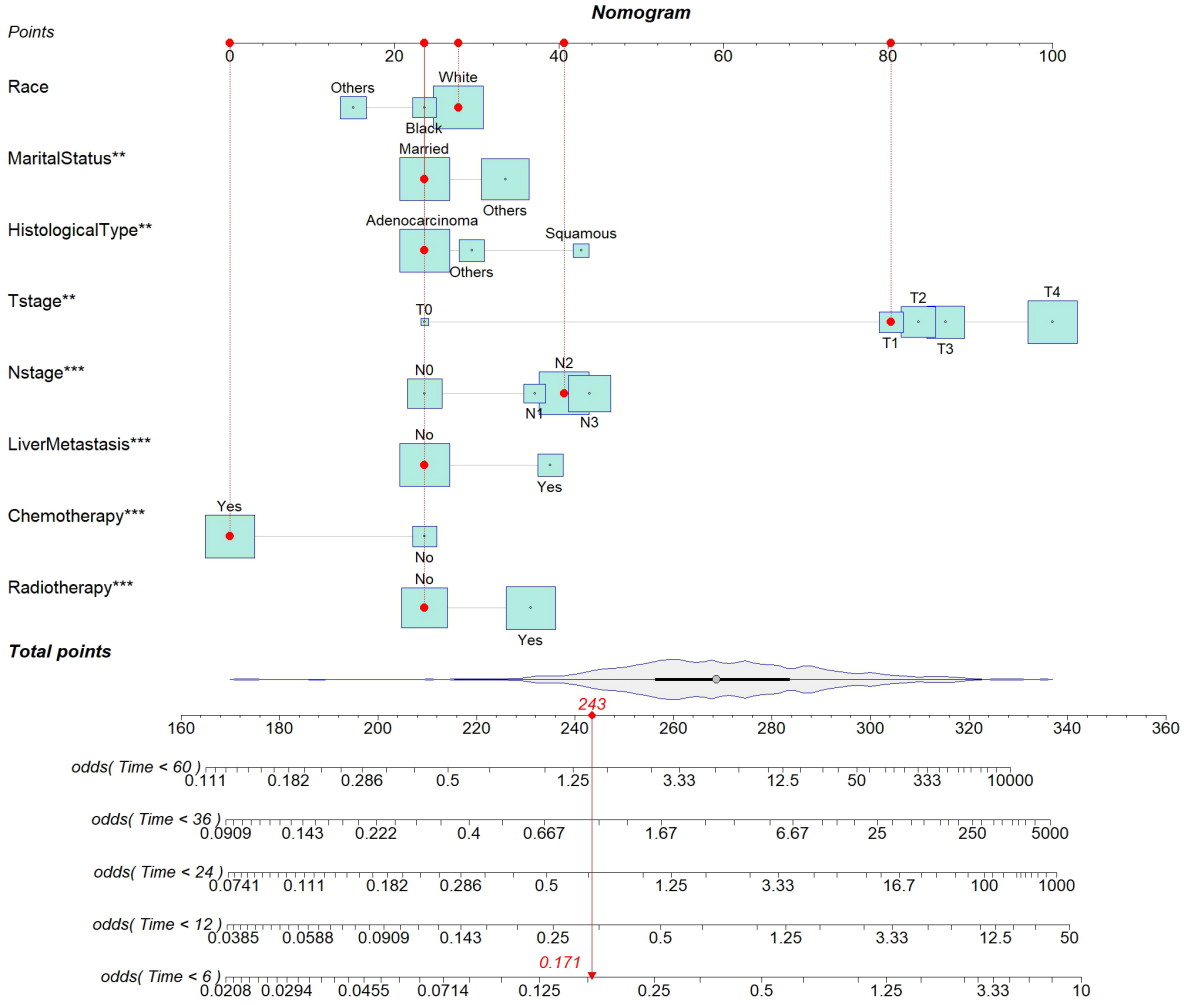
Nomogram for predicting the probability of 0.5-, 1-, 2-, 3-, and 5-year OS in young patients with metastatic NSCLC.

This study aimed to develop a nomogram model for young patients with metastatic NSCLC. Each risk factor was assigned a score, and the total score was summed by adding up these scores. Then, the OS rates at 0.5, 1, 2, 3, and 5 years were estimated using this total score, which could be read off the calibration curve on the total score axis. According to the survival prediction plot generated, T stage is the most influential prognostic factor, while marital status has the least impact on prognosis (**Figure 3**).

### 3.5 Validation of the nomogram

**Figure 4:**
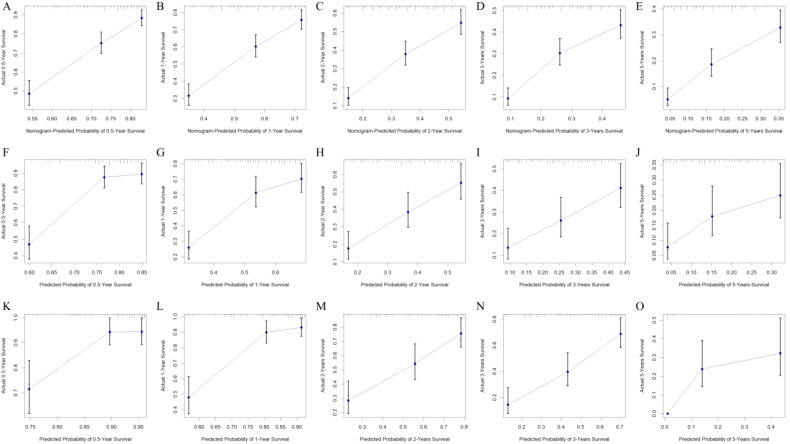
Calibration curves predicting the probability of 0.5-, 1-, 2-, 3-, and 5-year OS in the training **(A**–**E)**, internal validation **(F**–**J)**, and external validation cohorts **(K–O)**.

The nomogram underwent validation using both an internal (*n* = 288) and an external (*n* = 215) validation cohort. Calibration curves were then created for the training, internal validation, and external validation cohorts to evaluate the consistency between predicted and observed OS probabilities at intervals of 0.5, 1, 2, 3, and 5 years (**Figure 4**). These results demonstrate strong consistency across all cohorts, confirming robust predictive value of this nomogram.

**Figure 5:**
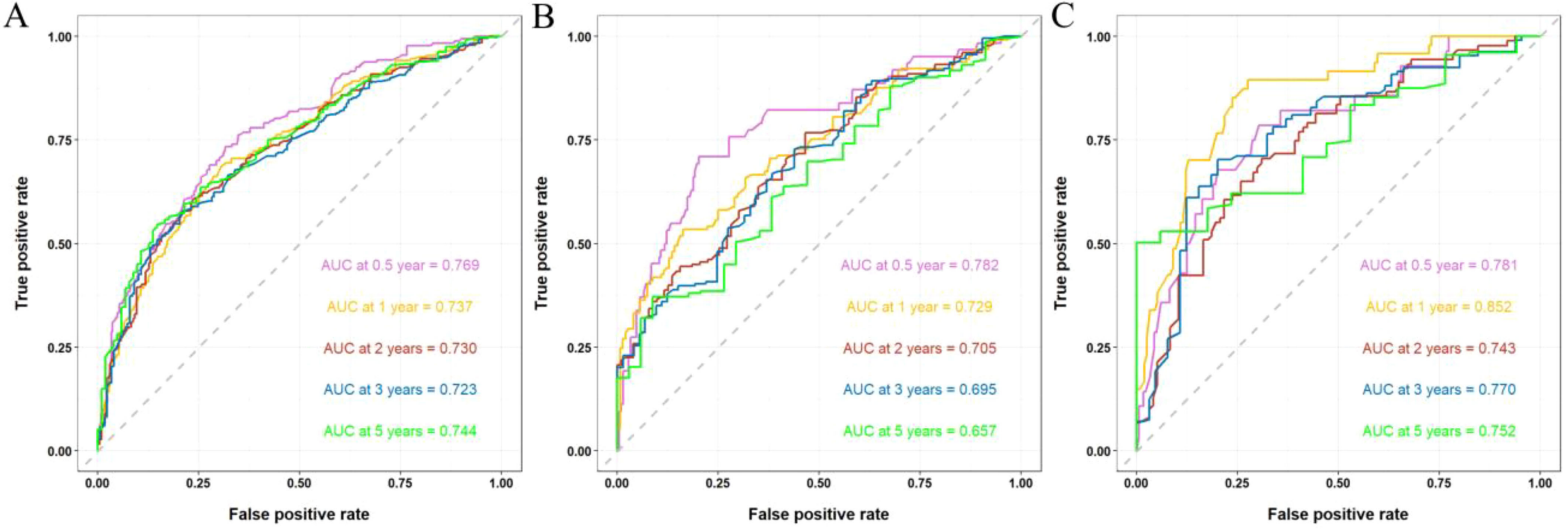
ROC curves and AUCs at 0.5-, 1-, 2-, 3-, and 5-year in the training **(A)**, internal validation **(B)**, and external validation cohorts **(C)**.

The discriminative ability of the nomogram was assessed using C-index and AUC metrics. In the training cohort, the C-index was 0.673 (95% CI = 0.661–0.685), with 0.5-, 1-, 2-, 3-, and 5-year AUCs of 0.769, 0.737, 0.730, 0.723, and 0.744, respectively. The internal validation cohort showed a C-index of 0.662 (95% CI = 0.643–0.681), and the 0.5-, 1-, 2-, 3-, and 5-year AUCs were 0.782, 0.729, 0.705, 0.695, and 0.657, respectively. The external validation cohort showed a C-index of 0.724 (95% CI = 0.702–0.746), and the 0.5-, 1-, 2-, 3-, and 5-year AUCs were 0.781, 0.852, 0.743, 0.770, and 0.752, respectively, demonstrating substantial predictive accuracy across all cohorts (**Figure 5**).

### 3.6 DCA curves

**Figure 6:**
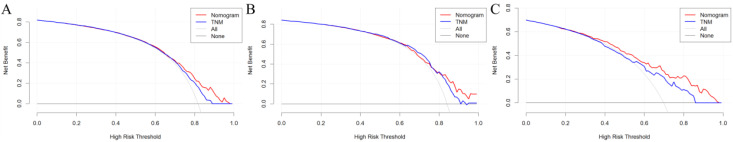
DCA of AJCC 7th TNM stage and nomogram for OS of the training **(A)**, internal validation **(B)**, and external validation cohorts **(C)**.

The DCA revealed that the nomogram exhibited a superior net benefit in predicting survival among young patients with metastatic NSCLC compared to AJCC TNM stage (**Figure 6**).

### 3.7 Survival analysis based on risk scores

**Figure 7:**
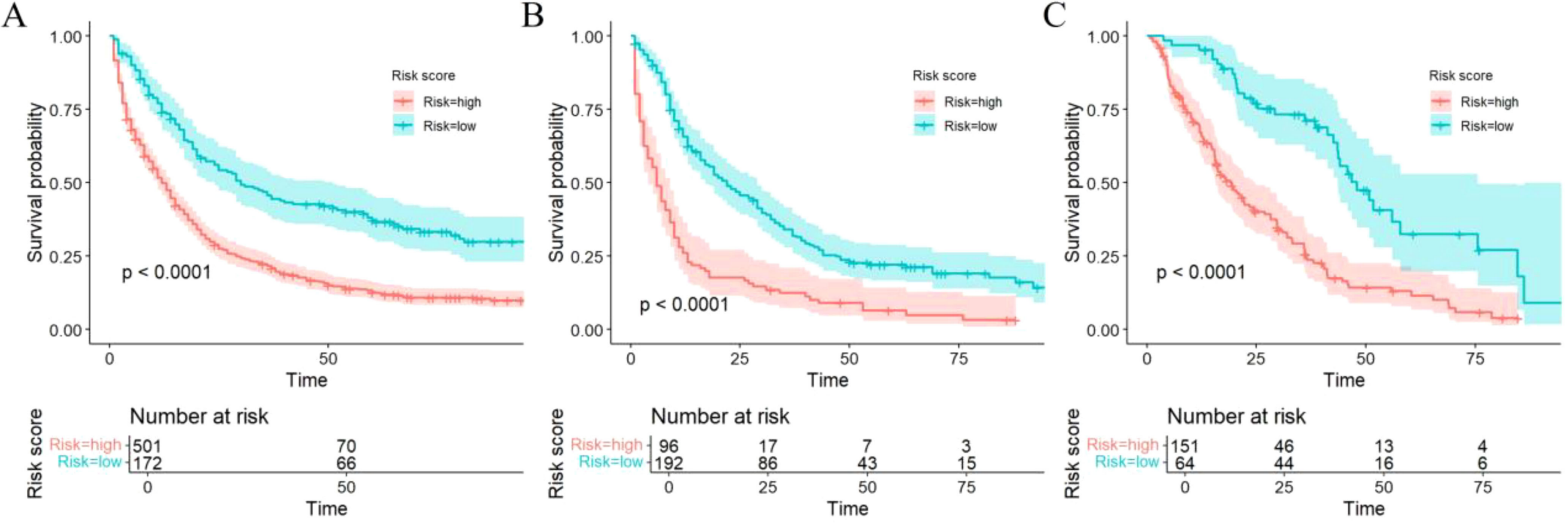
Kaplan–Meier curves of OS for risk stratification in the training **(A)**, internal validation **(B)**, and external validation cohorts **(C)**.

Patients were stratified into high-risk or low-risk group based on the risk scores obtained from the Cox proportional hazards regression model. Significant differences in survival were observed between the high and low-risk groups across the training, internal, and external cohorts, as demonstrated by Kaplan–Meier survival curves (all *P* < 0.0001) (**Figure 7**).

### 3.8 Optimization model with detailed clinical data and hematologic index

**Figure 8:**
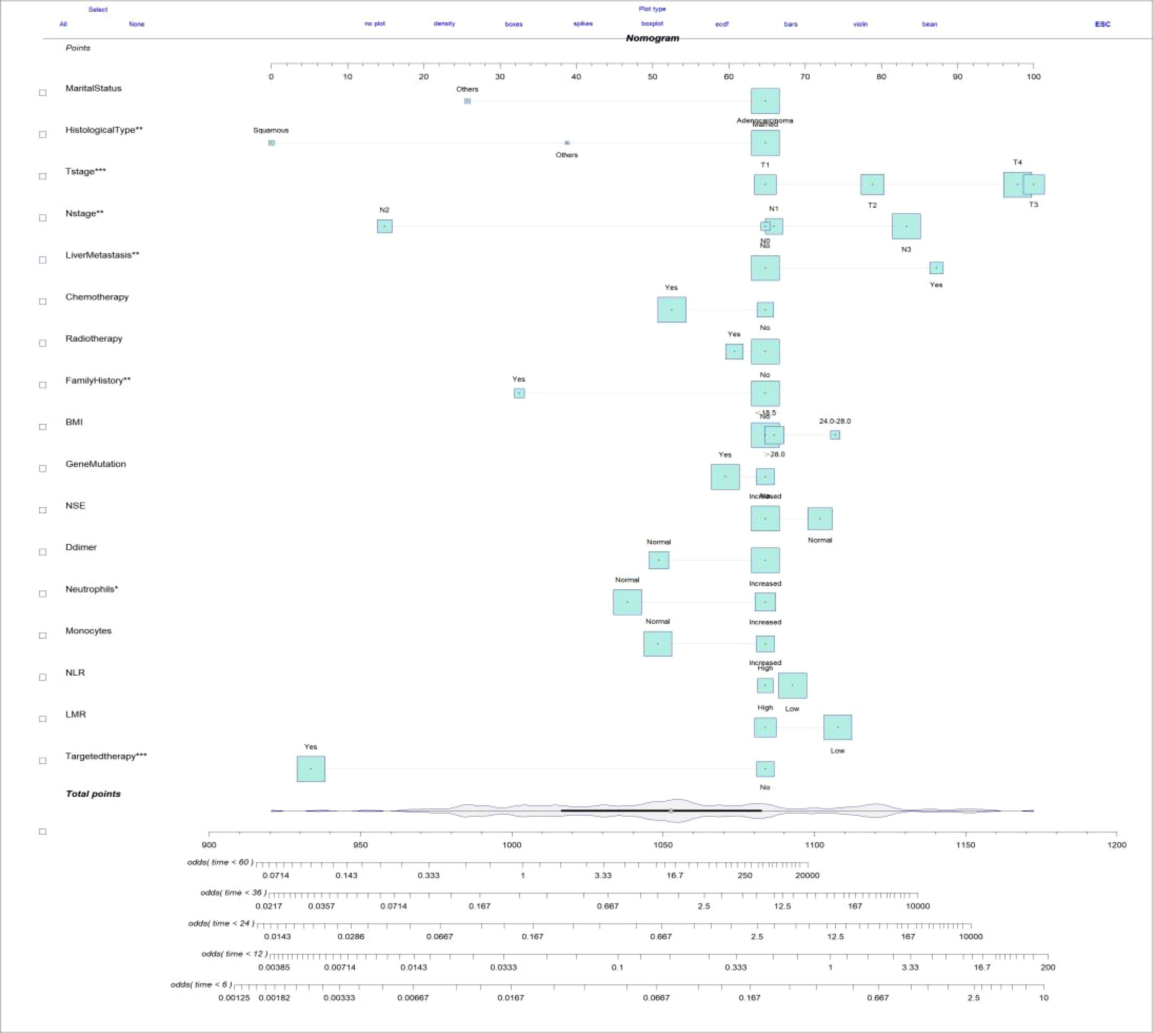
Optimized nomogram for predicting the probability of 0.5-, 1-, 2-, 3-, and 5-year OS in young patients with metastatic NSCLC.

**Figure 9:**
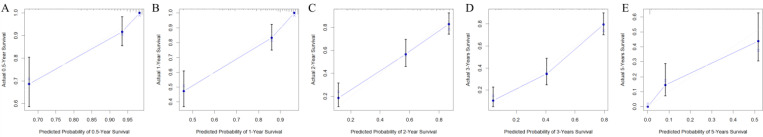
Calibration curves predicting the 0.5-, 1-, 2-, 3-, and 5-year OS of patients **(A–E)** after adding detailed clinical data and hematologic index.

**Figure 10:**
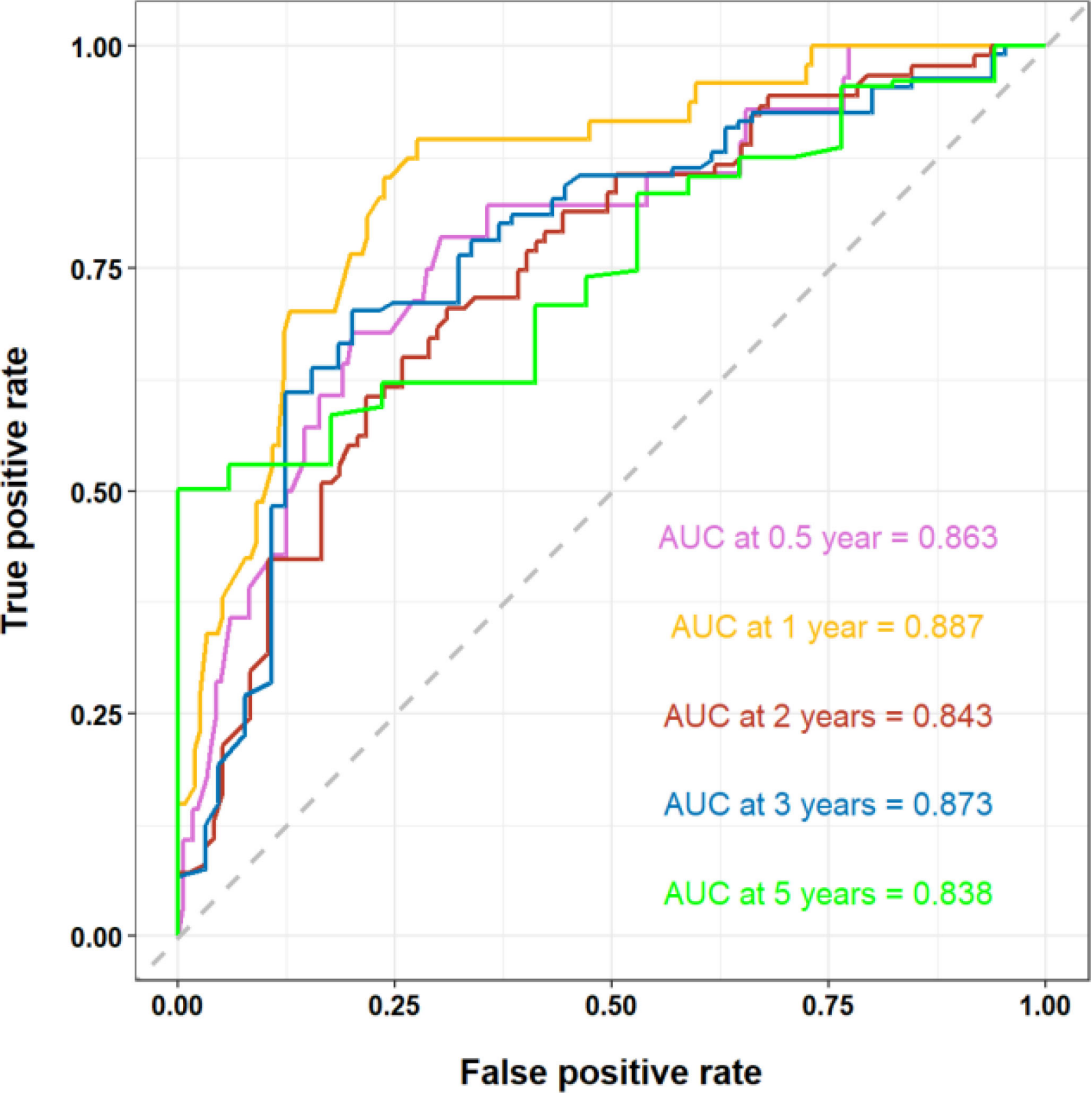
ROC curve after adding detailed clinical data and hematologic index.

**Figure 11:**
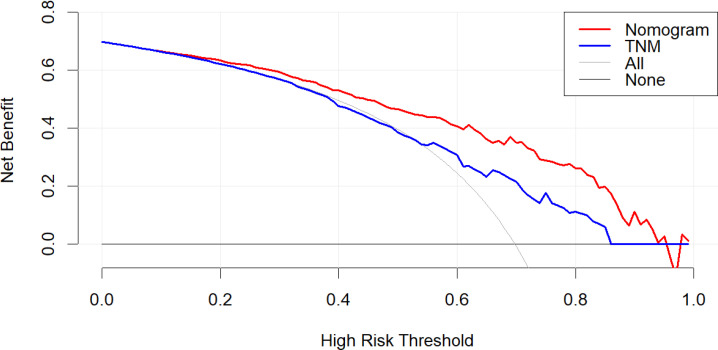
DCA curve after adding detailed clinical data and hematologic index.

The clinicopathologic data as well as treatment details of young metastatic NSCLC patients diagnosed at the Fourth Hospital of Hebei Medical University from 2013 to 2023 were analyzed. The external validation cohort included patients diagnosed with young NSCLC at the Fourth Hospital of Hebei Medical University between January 2013 and December 2023, with follow-up until July 31, 2024. Among them, 19 cases were lost to follow-up, and the follow-up rate was 91.2%. Univariate Cox regression analysis revealed several statistically significant predictors for survival (*P* < 0.05), including age, gender, marital status, smoking history, drinking history, family history, BMI, gene mutation, TNM staging, CEA, CYFBA, NSE, SCC, D-dimer, white blood cells, neutrophils, lymphocytes, monocytes, red blood cells, hemoglobin, platelets, NLR, LMR, PLR, bone metastasis, brain metastasis, liver metastasis, lung metastasis, chemotherapy, radiotherapy, immunotherapy, and targeted therapy. After Cox regression analysis, factors with *P* < 0.05 (family history, BMI, gene mutation, NSE, D-dimer, neutrophils, monocytes, NLR, LMR, and targeted therapy) were included in the optimized nomogram (**Figure 8**). The model showed a C-index of 0.796 (95% CI = 0.778–0.814). The calibration curves confirmed its accuracy (**Figure 9**). The AUC values for 0.5, 1, 2, 3, and 5 years were 0.863, 0.887, 0.843, 0.873, and 0.838, respectively, indicating superior discriminatory ability compared to the initial nomogram (**Figure 10**). The DCA curve showed that the optimized nomogram could achieve higher net gains (**Figure 11**).

## 4 Discussion

NSCLC in youth is a low-incidence-rate malignancy. First, given the distinct epidemiological characteristics of young metastatic NSCLC patients compared to elderly patients and the limitations of the TNM staging system, it is imperative to utilize a nomogram in establishing a predictive model for risk assessment and survival prognosis. Second, given the relatively small population of young lung cancer patients, we utilized the SEER database to ensure objectivity and credibility in our study. Furthermore, considering variations in ethnicity and genetic background across different populations, we established a Chinese external validation cohort based on our hospital data to validate the model and identify more effective predictive factors and indicators that are more clinically accessible than those available in the SEER database.

In this study, adenocarcinoma accounted for 73.4% of all cases among the patient cohort, indicating a significant majority, with a median survival time of 17 months. The OS rates at 0.5, 1, 2, 3, and 5 years were recorded at 78.3%, 62.1%, 40.3%, 29.5%, and 16.0%, respectively. In contrast, patients diagnosed with squamous cell carcinoma exhibited a median survival time of only 7 months; their corresponding OS rates at the same intervals were found to be 54.7%, 25.3%, 13.3%, 10.7%, and 5.3% (HR = 2.052, *P* < 0.001). This study underscores that the prognosis for lung adenocarcinoma is significantly more favorable than that for lung squamous cell carcinoma, aligning with previous research findings (15, 16). This may be attributed to a higher prevalence of sensitive gene mutations in adenocarcinoma patients, which provides them with enhanced opportunities for targeted therapy. Previous research has generally suggested that tumor size has a greater impact on the prognosis of early stage lung cancer patients. However, the current study showed that for metastatic NSCLC patients, both tumor size and lymph node metastasis status are independent factors influencing survival. In other words, survival outcomes in young metastatic NSCLC can still vary significantly based on their individual T and N stages. This indicates that even in the presence of distant metastasis, it is important to consider T and N stages when assessing patient prognosis. Liver metastasis, commonly associated with lower OS, occurs in up to 20% of advanced NSCLC cases (17, 18), with an incidence of 21.1% in the training cohort of this study. In this study, the 0.5-, 1-, 2-, 3-, and 5-year survival rate of patients with liver metastasis were 59.2%, 45.8%, 26.1%, 16.9%, and 7.7%, which was significantly lower than that of patients without liver metastasis (76.5%, 59.5%, 37.9, 29.8%, 18.1%) (HR = 1.519, *P* < 0.001). Young patients are at high risk of liver metastasis and have poorer prognosis (19, 20), potentially due to a more conducive angiogenic microenvironment that promotes tumor growth and metastasis (21).

Chemotherapy is crucial for controlling tumor growth and metastasis, playing a key role in treating advanced lung cancer. This study identified chemotherapy as an independent prognostic factor for young patients with metastatic NSCLC. Patients who received chemotherapy demonstrated significantly higher survival rates (*P* < 0.001). Furthermore, it has been noted that younger patients with lung cancer often exhibit a more favorable response to aggressive treatments such as chemotherapy and radiotherapy. This may be attributed to their better physical condition and ability to tolerate the side effects of these treatments. Furthermore, younger patients tend to have access to more advanced treatment options, which may significantly enhance their OS prognosis (22). Moreover, the superior enduring capacity of combination therapies in young lung cancer patients may also be ascribed to their stronger immune systems and ability to recover from the toxic effects of treatment. This resilience enables them to adhere to their treatment regimens without significant interruptions, ultimately leading to a higher likelihood of successful outcomes. Therefore, this has sparked our great interest in comparing whether adding local treatment to systemic treatment can improve the survival of late-stage young patients with NSCLC (16, 23). In this study, in young patients with metastatic NSCLC, adding local treatment to systemic treatment did not provide a survival benefit. In a subgroup analysis of the group receiving systemic therapy alone and the group receiving systemic therapy combined with local therapy, we found no significant difference in survival among young patients receiving systemic therapy (HR = 1.002, *P* = 0.064), regardless of whether local therapy was added. This suggests that local treatment of non-highly screened metastatic NSCLC did not provide a survival benefit. There are two potential explanations for this phenomenon. The first is that radiation therapy technology was largely considered outdated a decade ago, often accompanied by significant side effects that could diminish some of the survival advantages for patients. The second, and more critical, reason is that patients in the IV stage typically have a reduced survival duration; thus, it becomes imperative to rigorously screen candidates and prioritize those with limited metastasis—such as oligometastatic patients—for radiation therapy, which may underscore the efficacy of localized treatment.

Numerous studies have consistently demonstrated a strong correlation between inflammation and the onset as well as prognosis of tumors (24, 25). Neutrophils, lymphocytes, and monocytes play pivotal roles in the inflammatory response (26), with NLR and LMR serving as hematological indicators reflecting the body’s immune status. Elevated NLR values are associated with tumor aggressiveness (27), while increased LMR values indicate enhanced lymphocyte numbers and/or reduced monocyte counts, potentially leading to heightened tumor cell cytotoxicity and inhibition of tumor progression (28, 29). Incorporating survival-related variables and hematological measures from the external validation cohort into the nomogram improved the model’s predictive accuracy, as evidenced by higher C-index and AUC values. The C-index of the nomogram was 0.673, while that of the optimized model improved significantly to 0.796, indicating a substantial enhancement in predictive performance. The AUCs for the nomogram at 0.5-, 1-, 2-, 3-, and 5-year intervals were recorded as 0.769, 0.737, 0.730, 0.723, and 0.744, respectively; conversely, those for the optimized model were notably higher at 0.863, 0.887, 0.843, 0.873, and 0.838, demonstrating a progressive strengthening of predictive efficacy over time. This highlights the importance of further identifying prognostic factors and investigating straightforward, effective hematological markers to more accurately predict young patients with metastatic NSCLC at initial diagnosis.

To the best of our knowledge, this is not only the first nomogram established to predict the survival of young patients with metastatic NSCLC at initial diagnosis based on the SEER database, but it also validates the prediction of this nomogram model through internal and external cohorts. This study also found that in addition to the TNM staging system, hematological indicators also play an important role in predicting OS. However, there are some limitations to our study. First, the SEER database provided limited treatment details, such as chemotherapy regimens and radiation doses, and no genetic mutation information, which could have influenced our results. Second, the model only combines traditional therapeutic approaches and ignores newer ones, such as targeted therapy and immunotherapy. Finally, as a retrospective study, it inherently contains selection bias.

## 5 Conclusion

In summary, using the SEER database, this study found that independent prognostic factors in young patients with metastatic NSCLC included race, marital status, histological type, stage T, stage N, liver metastases, chemotherapy, and radiotherapy. Based on this, a prognostic prediction model was established for young patients with metastatic NSCLC at the time of initial diagnosis, which has been verified internally and externally with high prediction accuracy and can provide clinicians with appropriate treatment strategies and prognostic evaluation means.

## Data Availability

The raw data supporting the conclusions of this article will be made available by the authors, without undue reservation.
